# STAT1-induced upregulation of lncRNA KTN1-AS1 predicts poor prognosis and facilitates non-small cell lung cancer progression via miR-23b/DEPDC1 axis

**DOI:** 10.18632/aging.103191

**Published:** 2020-05-12

**Authors:** Changmin Liu, Xiaoming Li, Yanzhang Hao, Feng Wang, Zhiwen Cheng, Haitao Geng, Dianzhong Geng

**Affiliations:** 1 Department of Oncology, Binzhou Medical University Hospital, Binzhou, Shandong, China; 2 Department of Hepatobiliary Surgery, Shandong Provincial ENT Hospital, Shandong Provincial ENT Hospital Affiliated to Shandong University, Jinan, Shandong, China

**Keywords:** lncRNA KTN1-AS1, NSCLC, miR-23b, DEPDC1, metastasis

## Abstract

Several of the thousands of long noncoding RNAs (lncRNAs) have been functionally characterized in various tumors. In this study, we aimed to explore the function and possible molecular mechanism of lncRNA KTN1 antisense RNA 1 (KTN1-AS1) involved in non-small cell lung cancer (NSCLC). We identified a novel NSCLC-related lncRNA, KTN1 antisense RNA 1 (KTN1-AS1) which was demonstrated to be distinctly highly expressed in NSCLC. KTN1-AS1 upregulation was induced by STAT1. Clinical study also suggested that higher levels of KTN1-AS1 were associated with advanced clinical progression and a shorter five-year overall survival. Functionally, loss-of-function assays with in vitro and in vivo experiments revealed that KTN1-AS1 promoted the proliferation, migration, invasion and EMT progress of NSCLC cells, and suppressed apoptosis. Mechanistic studies indicated that miR-23b was a direct target of KTN1-AS1, which functioned as a ceRNA to subsequently facilitate miR-23b’s target gene DEPDC1 expression in NSCLC cells. Rescue experiments confirmed that KTN1-AS1 overexpression could increase the colony formation and migration ability suppressed by miR-23b upregulation in NSCLC cells. Overall, our findings imply that STAT1-induced upregulation of KTN1-AS1 display tumor-promotive roles in NSCLC progression via regulating miR-23b/DEPDC1 axis, suggesting that KTN1-AS1 may be a novel biomarker and therapeutic target for NSCLC patients.

## INTRODUCTION

Lung cancer is the most common cause of global cancer-related mortality, resulting in approximately 18.2 million new cases and 9.7 million deaths all over the world in 2018 [1]. Non-small cell lung cancer (NSCLC) is the most common type of lung cancer, accounting for 86% of all new cases [2]. In China, NSCLC ranked as the first healthy menace, with an increasing incidence and mortality in most provinces, especially in Guangdong [3]. Although over the last couple of decades mounting progresses have been achieved in the early screening and clinical treatments for this tumor, the 5-year survival rate for patients with advanced stages remains below 18% due to those patients often exhibiting distant metastasis and recurrence, which results in frequent failures of various treatment methods [4, 5]. Thus, the identification of novel mediators of metastasis and recurrence, in addition to sensitive markers of NSCLC progression, is essential for the improvement of the prognosis of patients.

Long non-coding RNAs (lncRNAs), a diverse type of long RNA molecules with lengths ranging from 200 nucleotides to 100 nucleotides, is considered as a “transcriptional noise” due to their limited protein-coding ability [6]. However, in recent years, the potential functions of lncRNAs as novel regulatory factors are receiving increased attentions [7]. More and more evidence revealed that lncRNAs display functional effects on many biological processes such as chromosome replication, cellular growth and differentiation via regulating gene expression at the transcriptional level or post-transcriptional level [8, 9]. In tumor progression, growing dysregulated lncRNAs are demonstrated to act as novel negative or positive regulators, which highlights that their potential clinical value used as therapeutic targets and tumor biomarkers [10, 11]. In recent years, several functional lncRNAs have been identified in NSCLC. For instance, lncRNA DLEU2 was reported to be highly expressed in NSCLC, and its knockdown suppressed the metastasis of tumor cells via regulating miRNA-30c-5p/SOX9 axis [12]. LncRNA LINC00657 was shown to promote the proliferation and metastasis of NSCLC cells by targeting miR-26b-5p/COMMD8 axis [13]. Although many studies have identified several lncRNAs in NSCLC, a large number of functional lncRNAs in NSCLC remained to be further identified and their completely clinical and basic roles and molecular mechanisms are still obscure.

KTN1 antisense RNA 1 (KTN1-AS1), a newly identified tumor-related lncRNA, was firstly reported to display high expressions in neck squamous cell carcinoma and predict unfavorable clinical outcomes [14]. Then, its overexpression and tumor-promotive roles were also confirmed in hepatocellular carcinoma and colorectal cancer [15, 16]. However, whether KTN1-AS1 was dysregulated in NSCLC, and its clinical significance as well as biological functions have not been investigated. In this research, our group firstly offered evidence that KTN1-AS1 was an overexpressed lncRNA in NSCLC, and then performed a series of clinical and functional assays. Our findings indicated that KTN1-AS1/miR-23b/DEPDC1 regulation axis may serve as a potential biomarker and therapeutic target for NSCLC.

## RESULTS

### KTN1-AS1 was highly expressed in NSCLC tumor specimens and predicted poor prognosis

**Figure 1:**
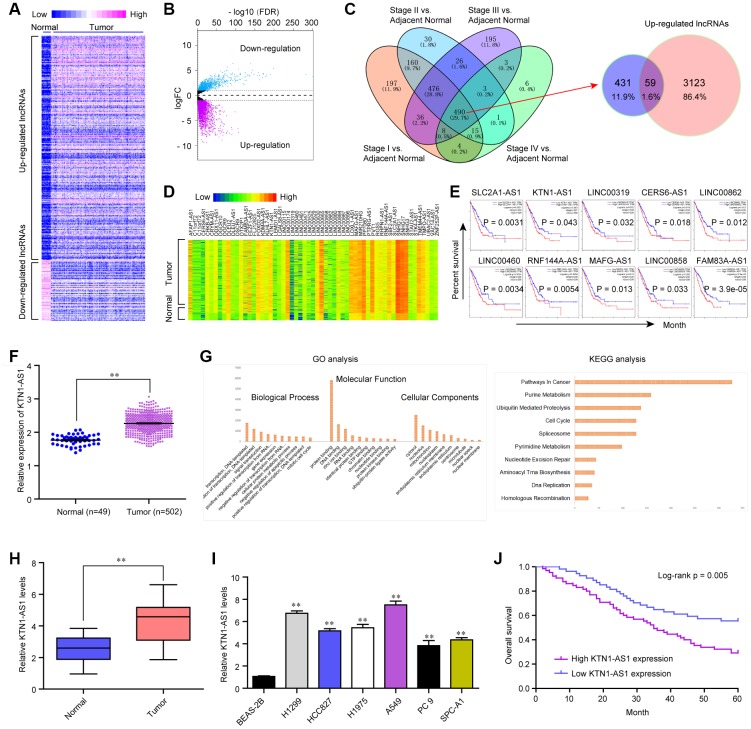
**KTN1-AS1 was up-regulated in NSCLC.** (**A**) Heatmap of differentially express (DE) lncRNAs using TCGA data analysis. (**B**) Volcano plots show differentially expressed lncRNAs based on the TCGA datasets. (**C**) Venn diagram of altered lncRNAs in TCGA datasets and the data from Cancer RNA-seq Nexus program. (**D**) The heatmap of the 59 lncRNAs expression which was analyzed above based on the TCGA datasets. (**E**) Overall survivals of several lncRNAs for NSCLC patients were analyzed by “GEPIA”. (**F**) Relative expression of KTN1-AS1 using TCGA data analysis. (**G**) GO and KEGG analysis for the preliminary exploration of KTN1-AS1 function. (**H**) qPCR analyzed the expression of KTN1-AS1 in our cohort. (**I**) Relative KTN1-AS1 levels in six NSCLC cells and BEAS-2B cells. (**J**) Kaplan-Meier survival analysis of NSCLC patients’ overall survival based on KTN1-AS1 expression in our cohort (n = 127). * P < 0.05, **P < 0.01.

To clarify the potential lncRNAs which played oncogenic roles in NSCLC, we first analyzed the TCGA dataset and found the differentially express (DE) lncRNAs in NSCLC tumor samples. The heatmap and volcano map of these DE lncRNAs were shown in Figure 1A, 1B. Afterwards, we searched Cancer RNA-seq Nexus program and obtained the lncRNAs which expressed in all stages (from stage I to IV) of NSCLC. These lncRNAs were intersected with the up-regulated lncRNAs in NSCLC tumor specimens which were analyzed using TCGA data, and 59 lncRNAs were found to be in both sides (Figure 1C). The expression (heatmap) of these 59 lncRNAs in NSCLC samples using TCGA data analysis was presented in Figure 1D. Thereafter, we searched GEPIA algorithm and found that only 10 of these 59 lncRNAs were significantly correlated with overall survival rates in NSCLC patients using TCGA data analysis (Figure 1E). Among these 10 lncRNAs, we next selected KTN1-AS1, a novel lncRNA, to further study. The levels of KTN1-AS1 in NSCLC samples were analyzed using TCGA data and KTN1-AS1 was remarkably up-regulated in NSCLC tumor tissues (Figure 1F). Subsequently, gene ontology (GO) and KEGG pathway analysis using Co-lncRNA program revealed that KTN1-AS1 was notably associated with pathways in cancers (Figure 1G). Next, qPCR was applied for detecting the levels of KTN1-AS1 in 127 NSCLC samples and KTN1-AS1 was also found to be markedly up-regulated in NSCLC tumor tissues (Figure 1H). In addition, the data from qPCR analyses suggested that lung cancer cells also expressed high KTN1-AS1 levels (Figure 1I). Then, we also explore the clinical significance of KTN1-AS1 in our cohort, finding that high expression of KTN1-AS1 was associated with TNM stage (*p* = 0.0029), Histological grade (*p*= 0.012) and lymph node metastasis (*p* = 0.020) (Table 1). Further survival assays also suggested that patients with high KTN1-AS1 expression had a shorter overall survival time than those with low KTN1-AS1 expression (*p* = 0.005, Figure 1J). More importantly, the results of multivariate assays suggested that KTN1-AS1 expression was an independent poor prognostic factor for five-year overall survival of NSCLC patients (HR=2.775, 95% CI: 1.282-4.219, *p* = 00021) (Table 2). Overall, the data indicated that KTN1-AS1 was highly expressed in NSCLC tumor specimens and predicted poor prognosis.

**Table 1:** Correlation between KTN1-AS1 expression and clinicopathological characteristics of NSCLC patients.

**Clinicopathological features**	**No. of cases**	**KTN1-AS1 expression**		***p* value**
		**Low**	**High**	
Age (years)				0.548
< 55	58	30	28	
≥ 55	69	32	37	
Sex				0.313
Male	70	37	33	
Female	57	25	32	
History of smoking				0.560
Ever	75	35	40	
Never	52	27	25	
Tumor size				0.209
≤ 3 cm	79	42	37	
> 3 cm	48	20	28	
TNM stage				0.029
I/II	80	45	35	
III/IV	47	17	30	
Histological grade				0.012
Well and moderately	76	44	32	
Poorly	51	18	33	
Lymph node metastasis				0.020
Negative	88	49	39	
Positive	39	13	26	

**Table 2:** Univariate and multivariate analyses for overall survival by Cox regression model.

**Parameters**	**Univariate analysis**			**Multivariate analysis**		
	**HR**	**95% CI**	***p***	**HR**	**95% CI**	***p***
Age	0.985	0.567-1.986	0.218	-	-	-
Sex	1.215	0.774-2.261	0.159	-	-	-
History of smoking	1.195	0.672-1.876	0.371	-	-	-
Tumor size	1.462	0.786-2.331	0.158	-	-	-
TNM stage	3.215	1.364-4.776	0.011	2.986	1.185-4.427	0.019
Histological grade	3.018	1.482-5.019	0.007	2.775	1.285-4.465	0.022
Lymph node metastasis	3.391	1.421-4.886	0.005	3.016	1.218-4.562	0.014
KTN1-AS1 expression	2.896	1.527-4.652	0.014	2.775	1.282-4.219	0.021

### STAT1 bound to KTN1-AS1 promoter and stimulated its expression in NSCLC cells

**Figure 2:**
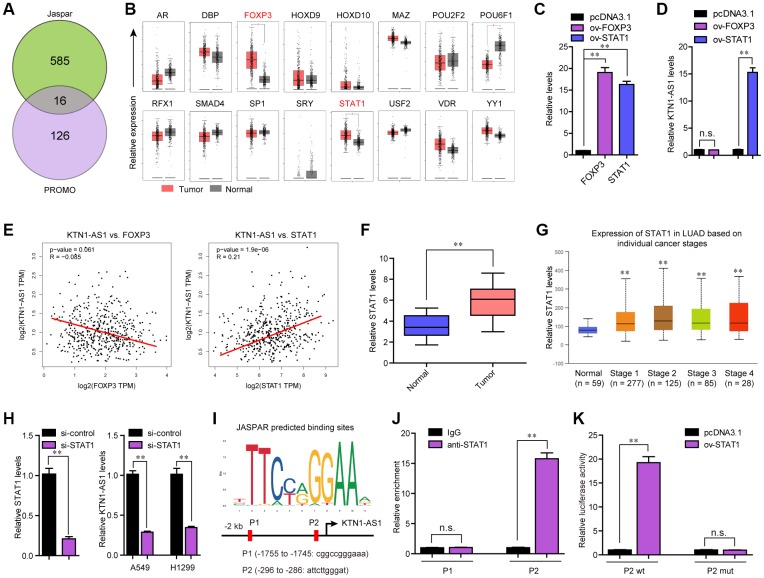
**STAT1 stimulate KTN1-AS1 dysregulation in NSCLC.** (**A**) Potential TFs were predicted by Jaspar and PROMO. The results of Jaspar and PROMO were intersected. (**B**) Expression of 16 TFs were analyzed by GEPIA using TCGA data. (**C** and **D**) qPCR detection for the determination of the association between overexpression of STAT1 and KTN1-AS1 expression. (**E**) The expressing correlation between KTN1-AS1 expression and FOXP3 and STAT1 expression was analyzed by GEPIA. (**F**) qPCR detected STAT1 expression in 127 NSCLC samples. (**G**) STAT1 expression across stages were analyzed by “UALCAN”. (**H**) KTN1-AS1 expression in A549 and H1299 cells after knockdown of STAT1 was determined by qRT-PCR. (**I**) Jaspar predicting potential binding sites between STAT1 and KTN1-AS1 promoter. (**J**) ChIP assay was performed to determine the affinity of STAT1 to KTN1-AS1 promoter. (**K**) Luciferase activity assays were applied to further confirm the binding of STAT1 to KTN1-AS1 promoter. * P < 0.05, **P < 0.01.

Considering that KTN1-AS1 was up-regulated in NSCLC tumor samples, we next attempted to uncover the potential mechanism which activated KTN1-AS1 aberrant dysregulation. Accumulating evidences had demonstrated that transcriptional factors (TFs) might promote lncRNAs transcription via directly binding to their promoters [17]. Therefore, we first obtained the KTN1-AS1 promoter sequence using UCSC website, and then applied Jarspar and PROMO programs to predict the possible TFs. We intersected the results of Jarspar and PROMO programs, and found only 16 TFs in both sides (Figure 2A). Then, we employed GEPIA program to analyze the expression of these 16 TFs using TCGA data, and found that only FOXP3 and STAT1 were significantly highly expressed in lung cancer specimens (Figure 2B). Thereafter, the FOXP3 and STAT1 overexpressing plasmids were respectively constructed, and transfected into A549 cells (Figure 2C). qPCR analysis demonstrated that only STAT1 but not FOXP3 could notably stimulate KTN1-AS1 expression (Figure 2D). Additionally, the correlation analysis from GEPIA program revealed that only STAT1 but not FOXP3 was positively correlated with KTN1-AS1 (Figure 2E). Therefore, we next focused on STAT1, and sought to investigate whether STAT1 was able to exactly bind to KTN1-AS1 promoter and induce KTN1-AS1 aberrant expression. Moreover, qPCR indicated that the levels of STAT1 were remarkably up-regulated in 127 NSCLC tumor samples (Figure 2F). Besides, the STAT1 levels were also up-regulated across stage I to IV of NSCLC tumor samples using UALCAN program analysis (Figure 2G). Subsequently, repressing the STAT1 levels was capable to reduce the KTN1-AS1 levels (Figure 2H). The Jaspar algorithm predicting binding sites were presented in Figure 2I. Additionally, we performed ChIP analysis and the data demonstrated that STAT1 could interact with P2 site of KTN1-AS1 promoter (Figure 2J). Moreover, luciferase activity detection assays validated that the luciferase reporter containing wild-type but not mutant-type P2 site obviously increased the luciferase activity in A549 cells with STAT1 ectopic expression (Figure 2K). In conclusion, our findings demonstrated that STAT1 activated the aberrant transcription of KTN1-AS1 in NSCLC.

### KTN1-AS1 inhibition suppressed cell proliferation and induced cell apoptosis *in vitro*

**Figure 3:**
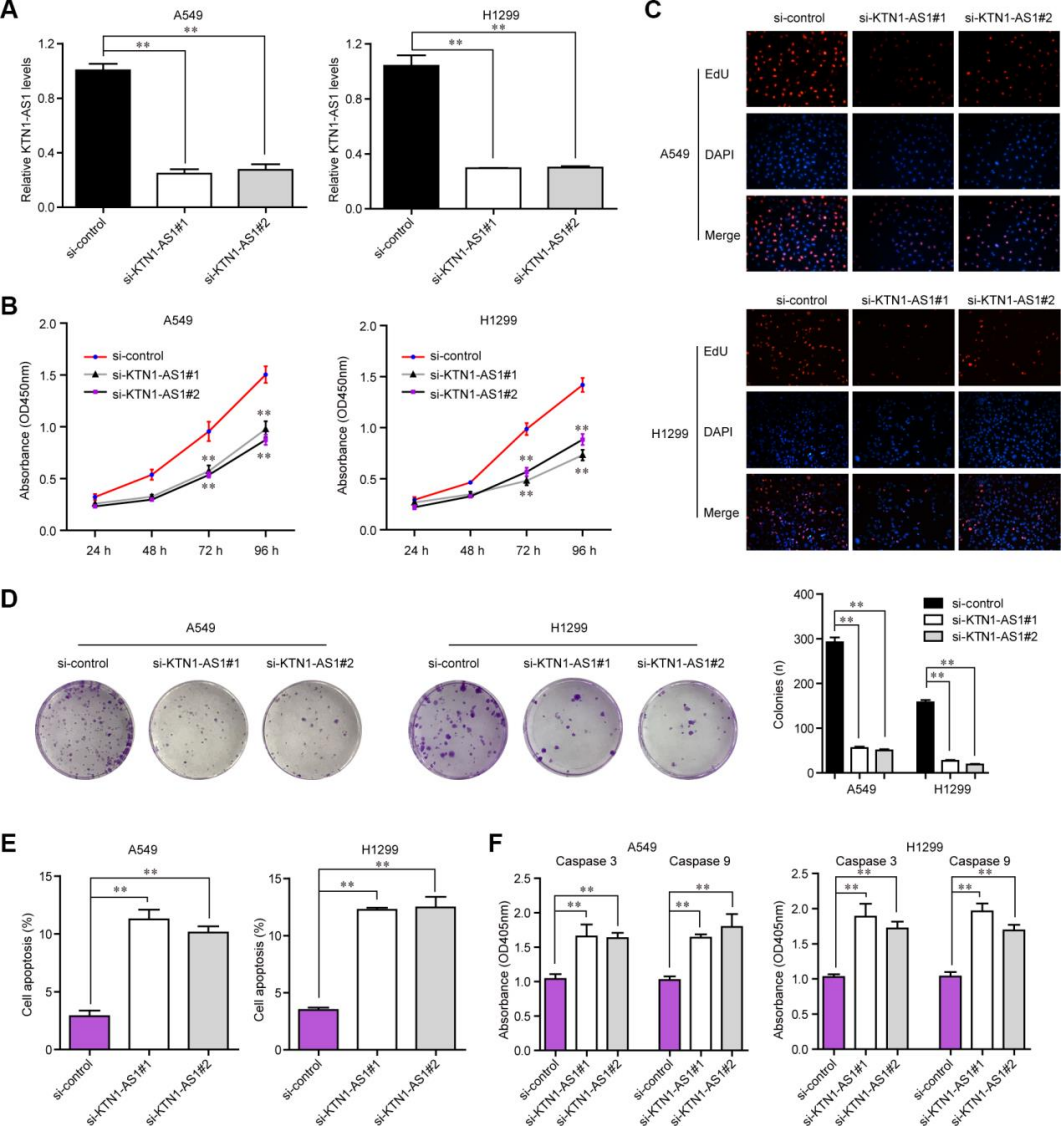
**KTN1-AS1 affected cell proliferation and apoptosis.** (**A**) qRT-PCR analysis of the expression of KTN1-AS1 in A549 and H1299 cell lines after si-KTN1-AS1. (**B**) The proliferation vitality detected using the CCK-8 assay. (**C**) EdU assays fro the determination of the cell proliferation ability of si-KTN1-AS1 transfected cells. (**D**) Colony formation assays for the functional exploration of knockdown of KTN1-AS1 on cellular growth. (**E**) The cells apoptosis was analyzed by flow cytometry. (**F**) The activities of caspase 3/9 were determined. * P < 0.05, **P < 0.01.

To explore the influence of altered KTN1-AS1 expression on NSCLC cells, we first performed qPCR to verify the knockdown efficiency of si-KTN1-AS1#1 and si-KTN1-AS1#2 in lung cancer cells, and the results suggested that these siRNAs had high knockdown efficiency for silencing KTN1-AS1 expression (Figure 3A). Then, we conducted CCK-8 assays to evaluate the influence of KTN1-AS1 depletion on cellular growth. The data clarified that KTN1-AS1 deficiency led to remarkable inhibition of lung cancer cell proliferation (Figure 3B). Similarly, the EdU assays also revealed that the proliferation of lung cancer cells was markedly decreased after KTN1-AS1 expression was silenced (Figure 3C). Furthermore, clonogenic assays also demonstrated that the colony formation abilities of lung cancer cells were notably suppressed after KTN1-AS1 was knocked down (Figure 3D). In addition, the effects of changed KTN1-AS1 expression on cellular apoptosis were also evaluated, and the results indicated that KTN1-AS1 depletion induced markedly cell apoptosis (Figure 3E). Mechanically, the caspase 3/9 activities in lung cancer cells after KTN1-AS1 depletion were determined, and we found that silencing KTN1-AS1 expression caused significantly increased caspase 3/9 activities (Figure 3F). These findings validated that the cell proliferation was suppressed, while the apoptosis was enhanced in KTN1-AS1-deficiency lung cancer cells.

### KTN1-AS1 deficiency inhibited tumor growth *in vivo*

**Figure 4:**
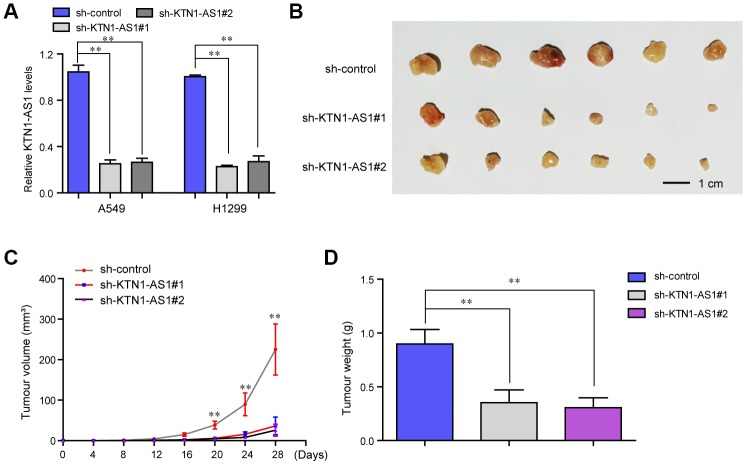
***In vivo* mice studies validated that KTN1-AS1 depletion suppressed tumor growth.** (**A**) Relative expression of KTN1-AS1 in A549 and H1299 cells transfected with sh-KTN1-AS1 (sh-KTN1-AS1 #1 or sh-KTN1-AS1 #2) and scrambled shRNA. (**B**) The photographs and comparison of excised tumor sizes in A549 cells. (**C**) The tumor volume-time curves. (**D**) The tumor weights. * P < 0.05, **P < 0.01.

To explore the effects KTN1-AS1 on lung cancer tumorigenicity *in vivo*, we established xenograft tumor models by using the A549 cells in nude mice. We first generated recombinant lentivirus carrying shRNA targeting KTN1-AS1 (sh-KTN1-AS1#1, sh-KTN1-AS1#2), and this lentivirus was then respectively used for infecting lung cancer cells. The qPCR analyses suggested that the lentivirus had high knockdown efficiency in lung cancer cells (Figure 4A). Subsequently, we subcutaneously injected A549 cells with KTN1-AS1 depletion into the nude mice. After four weeks, the mice were sacrificed and the weight of each tumor was measured. The tumors in each group were shown in Figure 4B. The results presented that the tumor volumes in the KTN1-AS1-deficiency groups were remarkably smaller than that in the sh-control group, indicating that repressing KTN1-AS1 expression could markedly abrogate the tumor growth (Figure 4C). Besides, the data suggested that lower tumor weights were found in the KTN1-AS1-depletion groups (Figure 4D). Taken together, these results demonstrated that KTN1-AS1 was crucial for NSCLC tumor growth *in vivo* and the findings further indicated that KTN1-AS1 was able to potentially serve as a new therapeutic target in NSCLC treatment.

### KTN1-AS1 knockdown impaired the mobility of NSCLC cells

**Figure 5:**
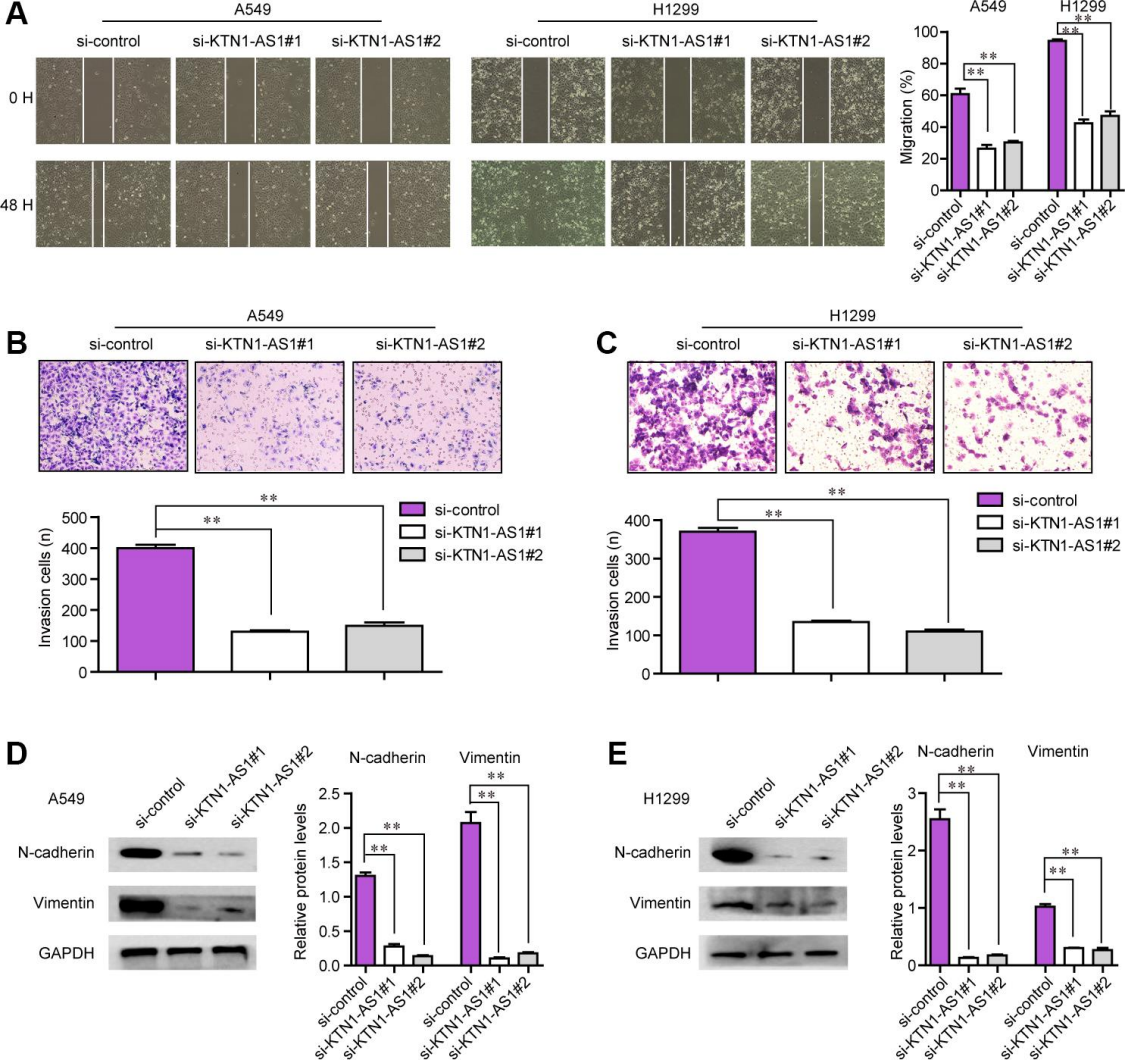
**The migration and invasion of NSCLC cells were regulated by KTN1-AS1.** (**A**) Cell migratory capabilities were assessed by wound healing assay after knocking down KTN1-AS1 in A549 and H1299 cells. (**B, C**) Cell invasive capabilities were examined by transwell assay after the knockdown of KTN1-AS1 A549 and H1299 cells. (**D**, **E**) western blot analyzed the protein levels of N-cadherin and vimentin. * P < 0.05, **P < 0.01.

The tumor cell invasion and migration were also major features that contributed to tumor development and progression. Therefore, we next attempted to explore whether KTN1-AS1 could modulate the metastatic potentials of NSCLC cells. To achieve that, wound-healing and transwell assays were conducted. The wound-healing assays presented that, within 48 h after transfection, NSCLC cells transfected with KTN1-AS1 siRNAs exhibited notably bigger gap distance than that treated with si-control, which indicated that KTN1-AS1 depletion caused significant inhibition of NSCLC cell migration (Figure 5A). Furthermore, transwell assays revealed that KTN1-AS1 siRNAs-transfected groups had a markedly smaller number of invaded cells when compared with the corresponding control group (Figure 5B, 5C). Since altered KTN1-AS1 expression influenced the metastatic capacities of NSCLC cells, we next sought to assess whether the levels of epithelial-to-mesenchymal (EMT) related molecules (N-cadherin and vimentin) were changed in NSCLC cells after KTN1-AS1 was knocked down. The data from western blot assays revealed that depression of KTN1-AS1 contributed to obvious suppression of N-cadherin and vimentin protein levels (Figure 5D, 5E). Collectively, our findings suggested that KTN1-AS1 depletion attenuated the metastatic potentials of NSCLC cells.

### KTN1-AS1 directly sponged miR-23b in NSCLC cells

**Figure 6:**
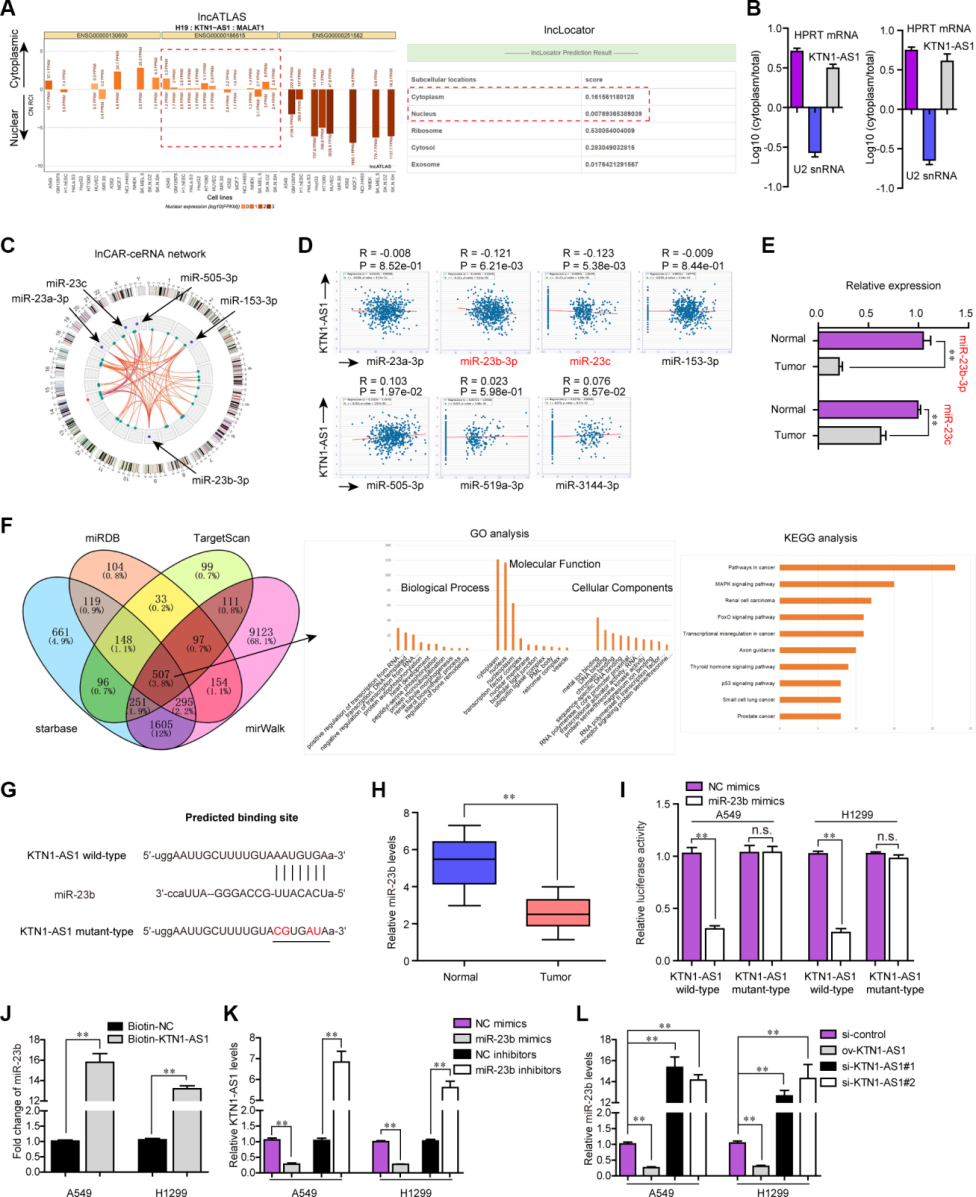
**miR-23b was directly targeted by KTN1-AS1.** (**A**) The subcellular localization of KTN1-AS1 was predicted by lncATLAS and lncLocator. (**B**) Subcellular fractionation assays. (**C**) The ceRNA network of KTN1-AS1 was analyzed by lnCAR. (**D**) starBase program analyzed the expressing correlation between KTN1-AS1 and miRNAs. (**E**) qPCR analysis detected the expression of miR-23b and miR-23c. (**F**) The intersection of the results from miRDB, TargetScan, starBase and miRWalk prediction. The commonly predicting genes were also used fo GO and KEGG analysis. (**G**) The predicting binding site between KTN1-AS1 and miR-23b. (H) qPCR assessed the miR-23b levels in 127 NSCLC tissues. (**I**) Relative luciferase activity detection. (**J**) RNA-pull down. (**K**, **L**) qPCR analysis detected the levels of KTN1-AS1 and miR-23b, respectively. * P < 0.05, **P < 0.01.

Afterwards, we sought to explore the mechanisms by which KTN1-AS1 served as crucial roles in NSCLC tumorigenesis. It is well known that lncRNAs, especially those exhibited in cytoplasm, exert their regulatory functions via acting as ceRNA [18]. Thereby, we first applied bioinformatics tools (lncATLAS and lncLocator) to predict the subcellular distribution of KTN1-AS1, and the results illustrated that KTN1-AS1 mainly distributed in cytoplasm (Figure 6A). Subsequently, the subcellular fractionation assays also validated that KTN1-AS1 mainly located in the cytoplasm of NSCLC cells (Figure 6B). Therefore, we next wondered whether any miRNAs might involve in KTN1-AS1 induced NSCLC tumorigenesis. To achieve that, bioinformatics analysis using lnCAR program was performed to construct the ceRNA network of KTN1-AS1, and five miRNAs (including miR-23a, miR-23b and miR-23c) were found to be in the network (Figure 6C). In addition, the starBase program was used for predicting the potential miRNA targets of KTN1-AS1 and among these miRNAs, the expression of only miR-23b and miR-23c was significantly (*P* < 0.01) negatively correlated with KTN1-AS1 expression in NSCLC specimens (Figure 6D). Hence, we next randomly selected three paired NSCLC tumor tissues and normal specimens to examine the levels of miR-23b and miR-23c, and the data from qPCR analysis showed that miR-23b levels were notably lower than that of miR-23c in NSCLC tumor tissues (Figure 6E). Therefore, we next sought to focus on miR-23b, a widely-reported tumor suppresser in diverse cancers. Indeed, GO and KEGG analysis of the common targets which were obtained by intersecting the results of miRDB, TargetScan, starBase and miRWalk, revealed that miR-23b was associated with pathways in cancer (Figure 6F). We thereby attempted to explore whether miR-23b was a target of KTN1-AS1 in NSCLC cells. The predicting binding sequence between KTN1-AS1 and miR-23b was presented in Figure 6G. Notably, qPCR analyses demonstrated that miR-23b levels were markedly decreased in 127 NSCLC tissues compared to normal samples (Figure 6H). Afterwards, the luciferase reporter assays were conducted and the results presented that miR-23b mimics obviously repressed the activity of KTN1-AS1 wild-type reporter plasmids, indicating that KTN1-AS1 directly sponged miR-23b (Figure 6I). Furthermore, RNA pulldown assays revealed that KTN1-AS1 could precipitate miR-23b in NSCLC cells (Figure 6J). Moreover, we also observed that ectopic expression of miR-23b notably suppressed KTN1-AS1 expression, while miR-23b depletion led to significantly increased KTN1-AS1 levels (Figure 6K). Similarly, qPCR analysis also indicated that KTN1-AS1 overexpression remarkably promoted miR-23b expression, while KTN1-AS1 deficiency caused markedly decreased KTN1-AS1 levels (Figure 6L). To sum up, the results validated that KTN1-AS1 was capable to sponge miR-23b to suppress its expression in NSCLC cells.

### DEPDC1 was a target of miR-23b in NSCLC cells

**Figure 7:**
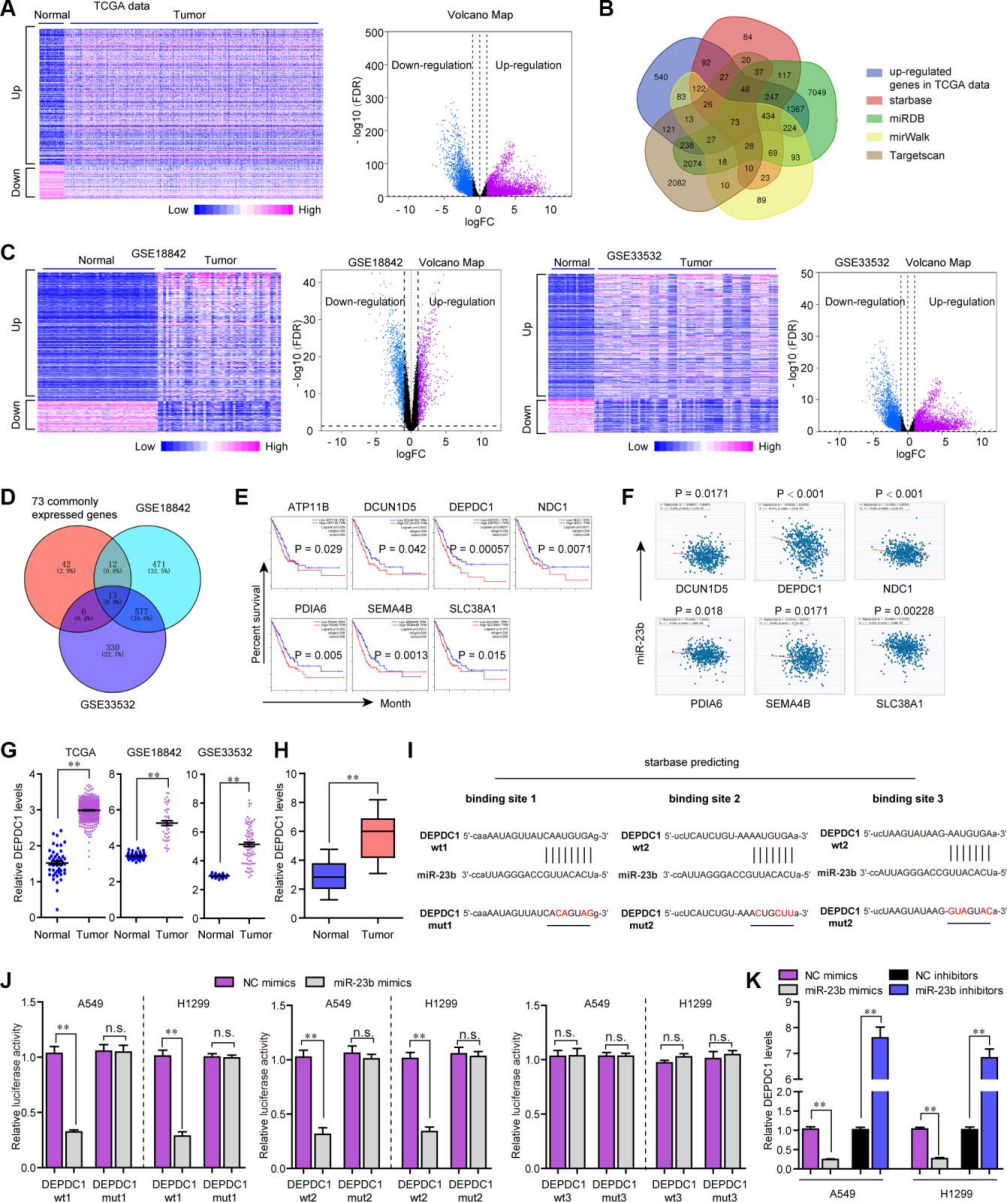
**MiR-23b directly targeted DEPDC1 in NSCLC cells.** (**A**) Heatmap and volcano map of DEGs using TCGA data analysis. (**B**) Veen diagram. (**C**) Heatmap and volcano map of DEGs using GSE18842 and GSE33532 data analysis. (**D**) Intersection of 73 commonly expressed genes and up-regulated genes in GSE18842 and GSE33532. (**E**) Overall survivals were analyzed by GEPIA. (**F**) starBase program analyzed the expressing correlation between miR-23b and 6 genes. (**G**) Relative levels of DEPDC1 in TCGA data, GSE18842 and GSE33532. (**H**) qPCR evaluated the DEPDC1 levels in 127 NSCLC tissues. (**I**) The three binding sites between miR-23b and 3’UTR of DEPDC1 mRNA were predicted by starBase program. (**J**) Luciferase activity detection. (**K**) qPCR evaluated the DEPDC1 levels in A549 and H1299 cells after transfection with miR-23b mimics and inhibitors. * P < 0.05, **P < 0.01.

MiRNAs are widely reported to modulate their downstream targets at post-transcriptional level. Therefore, we next aimed to uncover the potential target gene of miR-23b in NSCLC. For that purpose, we firstly conducted bioinformatics analysis to analyze the TCGA dataset and found the differentially express genes (DEGs) in NSCLC tumor specimens. The heatmap and volcano map of these DEGs were presented in Figure 7A. Thereafter, the up-regulated genes were intersected with the results of miRDB, TargetScan, starBase and miRWalk, and we obtained 73 common genes (Figure 7B). Besides, we analyzed DEGs in another two microarray profiles (GSE18842 and GSE33532), and the heatmaps and volcano maps were shown in Figure 7C. Afterwards, we intersected the above 73 common genes with the up-regulated genes in GSE18842 and GSE33532 data, and we found 13 commonly expressed genes (Figure 7D). Subsequently, GEPIA algorithm was used for analyzing the overall survivals of these 13 genes, and we found only 7 genes were significantly (*P* < 0.05) correlated with overall survivals (Figure 7E). Then, we conducted expressing correlation analysis using starBase program, and we found that, among these 7 genes, only six of them were significantly (*P* < 0.05) positively correlated with KTN1-AS1 expression in NSCLC samples (Figure 7F).

We then applied cBioportal program to analyze the genetic changes of these 6 genes and discovered that genetic alternation of DEPDC1, an extensively studied oncogene, was notable (Supplementary Figure 1A) [19]. GSCAlite algorithm analysis revealed that the expression and overall survivals across diverse cancer types of DEPDC1 were more obvious than other genes (Supplementary Figure 1B, 1C). The SNV frequency across cancers analyzed by GSCAlite algorithm also revealed that DEPDC1 had the most significant changes (Supplementary Figure 1D). Moreover, GSCAlite algorithm analysis also indicated that DEPDC1 was relevant with apoptosis, cell cycle and EMT (Supplementary Figure 1E). Besides, GSCAlite algorithm was also used to analyze the miRNA regulation network of these 6 genes, and we found that DEPDC1 was in the central miRNA regulation network (Supplementary Figure 1F). Therefore, we next attempted to focus on DEPDC1. The relative DEPDC1 expressing levels in TCGA data, GSE18842 and GSE33532 were presented in Figure 7G. Furthermore, UALCAN program analysis using TCGA data suggested that DEPDC1 was elevated across stage 1 to 4 of NSCLC tumor samples, and DEPDC1 was up-regulated in most cancer types (Supplementary Figure 2A, 2B). Afterwards, the genes positively correlated with DEPDC1 in lung cancer were obtained by using UALCAN program analysis, and GO and KEGG analysis revealed that DEPDC1 positively correlated genes were relevant with cell cycle (Supplementary Figure 2C, 2D). Hence, we next sought to investigate whether DEPDC1 was the target gene of miR-23b in NSCLC. qPCR assays also confirmed that DEPDC1 was up-regulation in 127 NSCLC tumor tissues (Figure 7H). The three binding sites between miR-23b and 3’UTR of DEPDC1 mRNA which were predicted by starBase program were shown in Figure 7I. Subsequently, we carried out the luciferase reporter assays, and the data certified that miR-23b mimics could remarkably repress the activities of DEPDC1 wt1 and wt2 reporters, while the activities of DEPDC1 wt3, DEPDC1 mut1, mut2 and mut3 were not changed, which indicated that miR-23b was directly interacted with DEPDC1 mRNA in NSCLC cells (Figure 7J). Besides, qPCR assays implied that miR-23b overexpression notably suppressed DEPDC1 expression, while miR-23b deficiency contributed markedly increased DEPDC1 levels (Figure 7K). Collectively, all the outcomes suggested that miR-23b directly targeted DEPDC1 in NSCLC.

### KTN1-AS1 modulated malignant behaviors of NSCLC via miR-23b/ DEPDC1 axis

**Figure 8:**
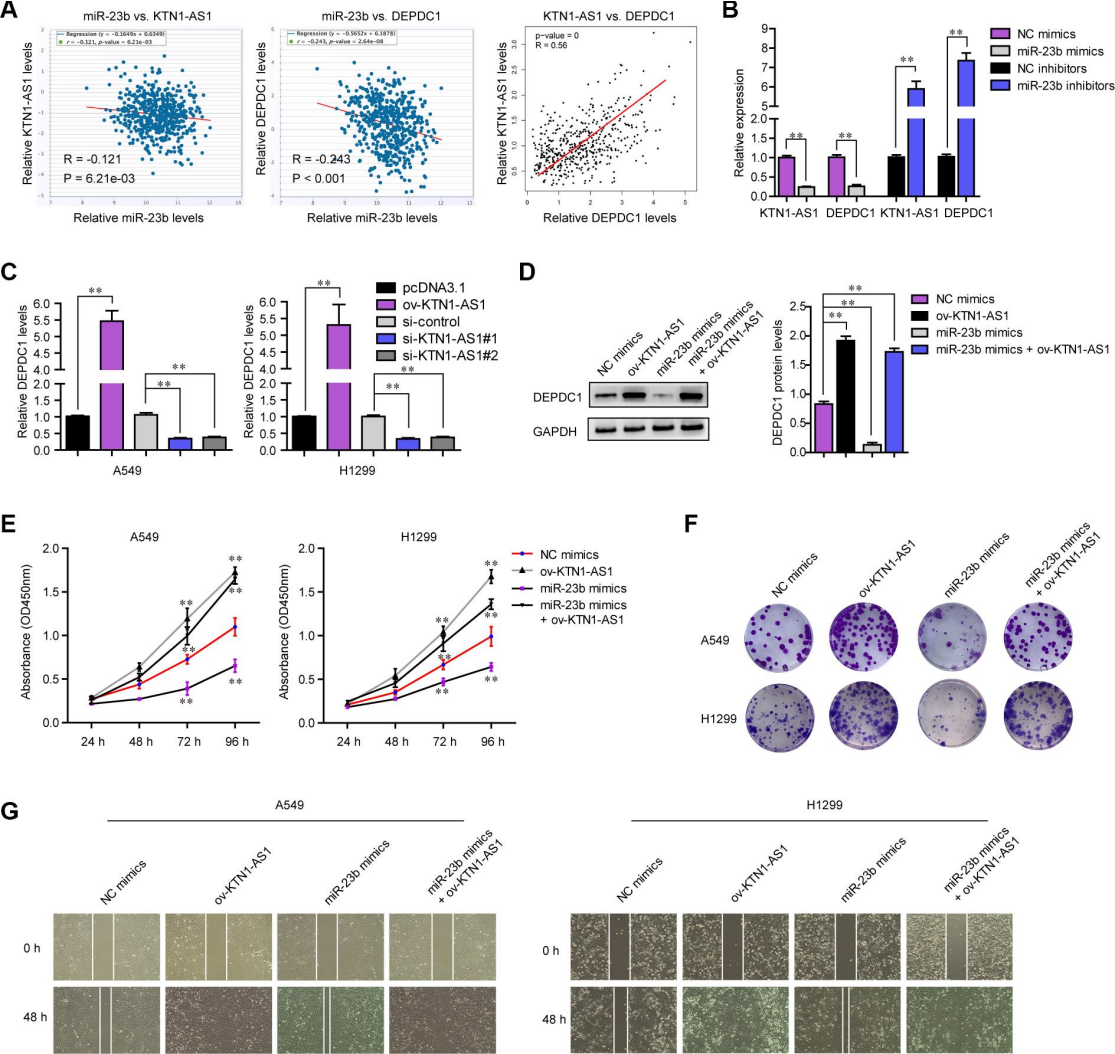
**KTN1-AS1 modulated DEPDC1 expression via miR-23b in NSCLC.** (**A**) starBase program analyzed the expressing correlation between miR-23b and KTN1-AS1, and DEPDC1. The expressing correlation between KTN1-AS1 and DEPDC1 was also analyzed by GEPIA. (**B**) qPCR evaluated the relative expression of KTN1-AS1 and DEPDC1 in A549 cells after transfection with miR-23b mimics and inhibitors. (**C**) qPCR examined the relative expression of DEPDC1 in NSCLC cells after their KTN1-AS1 was overexpressed or silenced. (**D**) Western blot determined the protein levels of DEPDC1 in A549 cells after various treatment. (**E**) CCK-8 assays determined the cellular growth. (**F**) Cell colony formation assays. (**G**) Wound-healing assays. * P < 0.05, **P < 0.01.

Next, the expressing relationships among KTN1-AS1, miR-23b and DEPDC1 were further investigated. Expressing correlation analysis using starBase and GEPIA algorithms revealed that miR-23b expression was negatively correlated with both KTN1-AS1 and DEPDC1, and KTN1-AS1 levels were positively correlated with DEPDC1 expression in NSCLC tumor specimens, which was consistent with the above observation that KTN1-AS1 sponged miR-23b and miR-23b directly targeted DEPDC1 (Figure 8A). In addition, qPCR analysis also clarified that forced expression of miR-23b caused significantly decreased levels of both KTN1-AS1 and DEPDC1, while repressing miR-23b expression led to significantly increased KTN1-AS1 and DEPDC1 expression (Figure 8B). Next, we sought to investigate whether KTN1-AS1 could directly modulate the expression of DEPDC1 in NSCLC cells. The results from qPCR assays demonstrated that overexpressing KTN1-AS1 (ov-KTN1-AS1) was capable to enhance DEPDC1 expression (Figure 8C). On the contrary, the DEPDC1 levels were depressed in lung cancer cells after their KTN1-AS1 was silenced. Afterwards, western blot was also conducted for evaluating the changes of DEPDC1 protein levels in A549 cells after treatment. The results confirmed that though miR-23b was capable to inhibit DEPDC1 expression, re-introduction of KTN1-AS1 was able to restore the protein levels which were suppressed by miR-23b mimics (Figure 8D). All these data demonstrated that KTN1-AS1 was capable to regulate DEPDC1 expression via targeting miR-23b. Next, we sought to investigate whether KTN1-AS1 could rescue the inhibitory effects of miR-23b on malignant behaviors of NSCLC cells. CCK-8 assays validated that enhancing KTN1-AS1 expression could significantly restore the cellular growth which was abrogated by transfecting miR-23b mimics (Figure 8E). Similar results that KTN1-AS1 overexpression rescued the colony formation abilities which were suppressed by miR-23b mimics, were also observed (Figure 8F). Although miR-23b was able to attenuate the migration of NSCLC cells, KTN1-AS1 overexpression could also restore the inhibitory influence of miR-23b on cellular migration (Figure 8G). Overall, these data suggested that KTN1-AS1 could modulate the malignancies of NSCLC via miR-23b/ DEPDC1 axis.

## DISCUSSION

Up to date, the clinical outcome of NSCLC patients remains poor in spite of the optimization of clinical treatments. In recent years, the developments of individualized treatments and precision medicines may greatly improve the long-term survival and quality of life of NSCLC patients [4, 20]. However, the lack of sensitive biomarkers limited the clinical application of novel targeted therapies. Recently, growing studies highlighted the potential application of lncRNAs used as novel sensitive biomarkers due to their presence in the plasma, good specificity, and accessibility [21, 22]. In this study, we performed a series of bioinformatics analysis and identified several possible functional lncRNAs in NSCLC. Importantly, a novel NSCLC-related lncRNA, KTN1-AS1, attracted our attention due to its distinct upregulation in NSCLC tissues and positive association with five-year overall survival of NSCLC patients. Then, clinical assays with 127 NSCLC patients demonstrated that higher levels of KTN1-AS1 were distinctly associated with advanced TNM stage, histological grade, positively lymph node metastasis and shorter 5-year overall survival, revealing that it may contribute to clinical progress of NSCLC patients. More importantly, based on the results of multivariate analyses, KTN1-AS1 was demonstrated to be an independent prognostic factor for NSCLC patients, which highlighted the clinical application of KTN1-AS1 used as a novel marker for NSCLC patients.

In recent years, more and more lncRNAs were demonstrated to be dysregulated in various tumors. However, the specific factors involved in their abnormally expressed pattern in tumor specimens remained largely unclear. Of note, growing evidence had indicated that some TFs and epigenetic modulators could display regulatory effects on the lncRNA transcription [23, 24]. For instance, overexpression of STAT1 increased the expressions of lncRNA LINC00174, resulting in the enhanced ability of cellular growth and metastasis in colorectal carcinoma [25]. SP1-induced up-regulation of lncRNA SNHG14 was demonstrated to promote the metastatic potential of cell renal cell carcinoma cells [26]. In this study, by the use of Jasper and PROMO tools, we identified 16 potential candidates for KTN1-AS1. Then, we searched “GEPIA”, finding that FOXP3 and STAT1 levels were distinctly upregulated in lung cancer. Moreover, only overexpression of STAT1 can promote the expression of KTN1-AS1. Thus, we chose STAT1 for our subsequent experiments. Previously, STAT1 had frequently reported to display functional effects on various tumor progression, and its overexpression could also activate the transcription of some lncRNAs in several tumors [25, 27, 28]. Based on ChIP and luciferase reporter assays, we observed that that STAT1 could bind to the KTN1-AS1 promoter region, thereby resulting in the transcription of KTN1-AS1. Overall, the above findings, together with previous researches, revealed that the dysregulation of TFs may display imperative functions on the overexpression of lncRNAs in tumors.

As a newly identified tumor-related lncRNA, only a few studies reported the expression pattern and functions of KTN1-AS1 in hepatocellular carcinoma and colorectal cancer. Kim et al. reported that KTN1-AS1 was up-regulation in colorectal cancer and distinctly promoted the cellular growth of colorectal cancer [16]. Zhang et al. showed that KTN1-AS1 expression was upregulated in hepatocellular carcinoma and predicted a shorter overall survival of hepatocellular carcinoma patients. Functionally, they demonstrated that knockdown of KTN1-AS1 inhibited the proliferation of hepatocellular carcinoma cells via regulating miRNA-23c/ERBB2IP axis [19]. However, whether KTN1-AS1 also displayed similar effects on NSCLC progression remains unknown. In this study, we firstly provided evidence that silence of KTN1-AS1 markedly attenuated the proliferation, migration and invasion of A549 and H1299 cells. In vivo assays also demonstrated the tumor-promotive roles of KTN1-AS1 in tumor growth. The EMT progress is a key event in promoting neoplasm tumor cells to migrate and invade [29]. Then, we further performed Western blot to examine whether knockdown of KTN1-AS1 could influence EMT progress, and the results revealed that the levels of N-cadherin and Vimentin were distinctly suppressed after the suppression of KTN1-AS1 using si-KTN1-AS1. Overall, our findings confirmed KTN1-AS1 as a tumor promoter in NSCLC cellular progression.

Salmena et al. firstly provided a novel hypothesis (ceRNA) to describe how different types of RNAs communicate with each other through miRNAs [30]. To study the potential mechanisms involved in the effects of KTN1-AS1 on the NSCLC progression, our group analyzed the localization of KTN1-AS1 in tumor cells due to its subcellular localization influencing the specific functions of KTN1-AS1 in cellular progress. Cytosolic lncRNAs were demonstrated to be involved in the modulation of mRNA stability and serve as regulators targeting miRNAs. From the results of the online tool, we observed that a larger proportion of KTN1-AS1 was observed in the nucleus, which was further confirmed by our cellular experiments. Using an online tool, we predicted several candidates for the possible targeting miRNAs of KTN1-AS1, including miR-23c, miR-23a-3p, miR-23b-3p, miR-505-3p and miR-153-3p. Moreover, the results of “ENCORI” revealed that there was a positive correlation between KTN1-AS1 and miR-23a-3p and miR-23c. Thus, we further chose the above two miRNAs for subsequent RT-PCR in the NSCLC tissues and matched normal specimens from our patients, finding that miR-23b-3p displayed lower expression in NSCLC specimens than non-tumor tissues. Therefore, our attention focused on miR-23b. The comprehensive application of four online databases showed 508 potential targeting genes of miR-23b. Using Go analysis and KEGG analysis to study the 508 genes, we observed that these genes were distinctly associated with the pathway in tumors, suggesting that miR-23b may be an important functional regulator in tumor progression. Actually, previous many studies have reported the dysregulation of miR-23b in various tumors, and this miRNA acted as a tumor suppressor in lung cancer [31, 32]. Interestingly, the results of RT-PCR revealed that the expressions of miR-23b were negatively regulated by KTN1-AS1 in NSCLC cells. Meanwhile, miR-23b also negatively modulated KTN1-AS1. Moreover, luciferase reporter assays and RNA pull-down assays both revealed that KTN1-AS1 directly targeted miR-23b in NSCLC cells. Our findings revealed that KTN1-AS1 may function as an endogenous sponge, suppressing both miR-23b expression and the functions of miR-23b.

It has been known to us that miRNAs displayed their tumor-promotive or suppressive roles via targeting different target genes. To investigate the miRNA-related functions of KTN1-AS1 in NSCLC progression, we chose miR-23b as a model miRNA for subsequent experiments. Based on the results of bioinformatics assays, we screen several possible potential targeting genes. Our attention focused on DEPDC1 which was demonstrated to be distinctly highly expressed in NSCLC tissues and predict a poor clinical outcome of NSCLC patients. DEPDC1, located on 1p31.3, was a newly discovered novel tumor-associated gene and highly conserved from Caenorhabditis elegans to human [33]. Overexpression of DEPDC1 and its oncogenic roles were frequently demonstrated in several tumors, such as hepatocellular carcinoma and prostate cancer [34, 35]. In NSCLC, a previous study by Wang et al. had reported that DEPDC1 was highly expressed, which was consistent with our results [19]. However, the mechanisms involved in overexpression of DEPDC1 in NSCLC remained unclear. Using luciferase reporter assays, DEPDC1 was demonstrated to be a direct target of miR-23b in A549 and H1299 cells. Further cellular experiments also confirmed overexpression of miR-23b decreased the expressions of DEPDC1 in both A549 and H1299 cells, while knockdown of miR-23b displayed an opposite function. These findings prompted us to investigate whether suppression of KTN1-AS1 exhibited its tumor-suppressive potential via modulation of DEPDC1 expression. In expressing levels, we confirmed that the overexpression of KTN1-AS1 could increase the mRNA and protein levels of DEPDC1 which was decreased by miR-23b mimics. Besides, functional assays also demonstrated that the forced upregulation of KTN1-AS1 could promote the tumor cellular proliferation and invasion suppressed by miR-23b mimics in NSCLC cells. Overall, our findings suggested that KTN1-AS1 exhibited its oncogenic effects at least in part by targeting miR-23b to modulate DEPDC1 expression.

Taken together, we identified a novel NSCLC-related lncRNA KTN1-AS1 which was increased in CRC cells and tissues, and was activated by transcription factor STAT1. We also confirmed that KTN1-AS1 could promote NSCLC progression as a ceRNA through sponging miR-23b and releasing DEPDC1. Therefore, KTN1-AS1/miR-23b/DEPDC1 may be a promising therapeutic target for NSCLC patients, enhancing the clinical benefits of targeted therapy.

## MATERIALS AND METHODS

### Tissue collection

One hundred and twenty-seven NSCLC tumor tissues and adjacent normal specimens were collected from patients who underwent surgery at Binzhou Medical University Hospital from June 2011 to October 2014. Before surgery, patients did not receive any anti-cancer treatment. After surgery, the samples were quickly frozen in liquid nitrogen. All excised specimens were examined by pathologists. The patients signed the written informed consents. The procedures were approved by the ethics committee of Binzhou Medical University Hospital.

### Cell transfection

NSCLC cells: H1299, HCC827, H1975, A549, PC 9 and SPC-A1 cells, were bought from CAS Shanghai Cell Bank (Xuhui, Shanghai, China). The control cells: BEAS-2B cells, were purchased from Kebai Biological company (Changsha, Hunan, China). RPMI-1640 media containing 10% FBS were used for culturing the cells. The cells were placed in an incubator at 37 °C with 5% CO_2_. The cell transfection was conducted by using Lipofectamine 3000 reagents (Fujun, Shenzhen, Guangdong, China) according to the kits’ protocols. SiRNAs targeting STAT1 (si-STAT1), KTN1-AS1 (si-KTN1-AS1#1, si-KTN1-AS1#2), miR-23b mimics, miR-23b inhibitors were purchased from Shanghai Shenggong Biological corporation (Songjiang, Shanghai, China). STAT1 or KTN1-AS1 was cloned into pcDNA3.1 empty vector to consistently express STAT1 (ov-STAT1) or KTN1-AS1 (ov-KTN1-AS1), respectively.

### Real-time PCR

Total RNAs were isolated from specimens and cells using TRIzol kits (YunBio, Shenzhen, Guangdong, China). Then, the RNA concentrations were determined by Nanodrop 2000 apparatus (Thermo Fischer Scientific, Waltham, MA, USA). Afterwards, cDNA was reversely transcribed using Takara PrimeScript kits (YunBio, Shenzhen, Guangdong, China). The qPCR analysis for KTN1-AS1, STAT1 and DEPDC1 were then conducted with Qiagen SYBR Green real-time PCR kits (Kehong, Xiamen, Fujian, China). We used GAPDH as an endogenous control for these mRNAs or lncRNA. For miRNA detection, Qiagen miRNeasy kits (Kehong, Xiamen, Fujian, China) were used to extract the miRNAs according to the kits’ instructions. Then, TransScript Green miRNA Two-Step qPCR kits (Quanshijin, Haidian, Beijing, China) were used for detecting the levels of miRNAs according to the protocols in the kits. The miRNA expression was normalized to U6 small nuclear RNA. The primers were listed in Table 3. The qRT-PCR results were analyzed with the 2^-ΔΔCt^ method.

**Table 3:** The primers used in this study for RT-PCR.

**Names**	**Sequences (5’-3’)**
KTN1-AS1: F	ATGCACACTTCTCGGCTAAGAGTC
KTN1-AS1: R	CTACAATGCCACAAGTGATTCCAGC
STAT1: F	CAGCTTGACTCAAAATTCCTGGA
STAT1: R	TGAAGATTACGCTTGCTTTTCCT
miR-23b: F	GCCCATTAGGGACCGTTA
miR-23b: R	CTCAACTGGTGTCGTGGA
DEPDC1: F	GGTGTGCCATCCCTAGAAGA
DEPDC1: R	CATTGCTTCTTGGCCAATTT
GAPDH: F	CAATGACCCCTTCATTGACC
GAPDH: R	GACAAGCTTCCCGTTCTCAG
U6: F	CTCGCTTCGGCAGCACA
U6: R	AACGCTTCACGAATTTGCGT

### Bioinformatics analysis

Microarray data were downloaded from TCGA dataset and GEO datasets (GSE18842 and GSE33532). The differentially expressed lncRNAs and genes in lung cancer specimens were analyzed by R packages. The heatmaps and volcano maps were also generated by R packages. The expression of the transcription factors was analyzed using GEPIA website (http://gepia.cancer-pku.cn/) or UALCAN program (http://ualcan.path.uab.edu/) [36, 37]. Gene correlation analysis was conducted by using GEPIA and starBase (http://starbase.sysu.edu.cn/). LncRNAs which expressed in all stages (from stage I to IV) of NSCLC were analyzed by Cancer RNA-seq Nexus (http://syslab4.nchu.edu.tw/) [38]. The potential binding sites between STAT1 and KTN1-AS1 promoter were predicted by Jaspar (http://jaspar.genereg.net/) or PROMO (http://alggen.lsi.upc.es/) [39]. The subcellular localization was predicted by lncATLAS (http://lncatlas.crg.eu/) and lncLocator (http://www.csbio.sjtu.edu.cn/bioinf/lncLocator) [40]. The target genes of miR-23b were predicted by miRDB (http://mirdb.org/), TargetScan (http://www.targetscan.org/vert_72/), starBase and miRWalk (http://mirwalk.umm.uni-heidelberg.de/). The overall survivals of corresponding lncRNAs and genes were generated by GEPIA. Gene ontology (GO) and KEGG analysis were conducted by DAVID program (https://david.ncifcrf.gov/home.jsp) or Co-LncRNA (http://bio-bigdata.hrbmu.edu.cn/Co-LncRNA/). The genetic alteration was analyzed by cBioportal (http://www.cbioportal.org/). The expression, survivals, SNV alteration, pathway activity and miRNA regulation network of gene sets were anal;yzed by GSCALite (http://bioinfo.life.hust.edu.cn/web/GSCALite/). The KTN1-AS1 ceRNA network was analyzed by lnCAR (https://lncar.renlab.org/).

### Cell growth evaluation

The cellular growth was examined by CCK-8 assays using CCK-8 detection kits (Jikai, Pudong, Shanghai, China). Briefly, twelve hours post-transfection, NSCLC cells were trypsinized, washed by PBS and then re-placed into ninety-six-well plates (2500 cells per well). After incubation, 15 μl CCK8 reagents were added into the wells at different time (24, 48, 72 and 96 h). Then, the plates were placed in an incubator (5% CO_2_, 37 °C) for 2.5 h. Finally, the absorbance (at OD450nm) was examined by a microplate reader apparatus.

### Clonogenic formation assay

After siRNAs transfection (24 h later), NSCLC cells were digested and collected in a centrifuge tube. After washing using PBS buffer twice, the cells were counted and subsequently placed into six-well plates (about 500 cells per well). The media (with 10% FBS) were changed every three days. After incubation for 14 days, methanol (1 ml) with crystal violet (0.1%) was used to treat the colonies for 15 min. After washing three times using PBS, the photographs of the colonies were taken by a camera.

### EdU assay

The EdU assay was also used for determining the cellular proliferation. The EdU assay kits were obtained from Guangzhou Ruibo Biological corporation (Guangzhou, Guangdong, China). Briefly, twenty-four hours post-transfection, NSCLC cells were digested and re-plated into forty-eight-well plates. The cells were then cultured for 24 h. Thereafter, 25 μl EdU reagents (final concentration: 10 μM) were placed into each well. The plates were then kept at 37 °C with 5%CO_2_ for 2.5 h. Afterwards, paraformaldehyde (4%) and Triton X100 (0.5 %) were used for treating the cells. Thereafter, Click-iT reactions were added into the plates and incubated for 25 min. After that, DAPI reagents were applied for staining the nuclei. Finally, a fluorescence microscope was used to image the fluorescence of the cells.

### Western blot

The NSCLC cells after KTN1-AS1 knockdown were lysed using lysis buffer (Kehong, Xiamen, Fujian, China). Afterwards, the BCA kits (Jingwei, Changsha, Hunan, China) were applied for determining the protein concentrations of the lysates in accordance with the kits’ protocols. Then, 30 μg total cellular proteins were resolved by SDS-PAGE electrophoresis (8-12%), followed by being electro-transferred to the PVDF membranes. Thereafter, the membranes were treated using 5% BSA solution (1 h, 27 °C), followed by being probed with primary antibodies against the following proteins: N-cadherin (1:900; Abcam, Cambridge, MA, USA), vimentin (1:800; CST, Danvers, MA, USA), GAPDH (1:2500; PTG, Wuhan, Hubei, China). The membranes were then respectively treated with the primary antibodies (12 h, 4 °C). Subsequently, the membranes were washed using TBST buffer, followed by being treated with a matched secondary antibody. Finally, the membranes were applied for presenting the protein bands by using ECL kits (Beyotime, Nantong, Jiangsu, China).

### Cell apoptosis detection

Annexin V-FITC Apoptosis detection kits (Beyotime, Nantong, Jiangsu, China) were employed for determining the apoptosis of NSCLC cells after transfection with KTN1-AS1 siRNAs. In brief, the lung cancer cells after treatment with KTN1-AS1 siRNAs were collected and subsequently re-placed into 1× binding buffer (500 μl). Then, the cellular suspensions were treated with 5 μl Annexin V-FITC, flowed by being added with 5 μl PI. After incubation for 25 min in the light-proof condition, the cells were washed, collected and re-suspended into 1× binding buffer (250 μl), followed by being examined using a flow cytometry machine.

### Caspase activity detection

Caspase activity detection kits (Beyotime, Nantong, Jiangsu, China) were used for assessing the activities of caspase 3/9 in KTN1-AS1-depleted NSCLC cells. Briefly, the cells after KTN1-AS1 knockdown were collected and washed by PBS. Subsequently, the collected cells were treated with Lysis Buffer (4°C, 35 min). Thereafter, the supernatants were collected by centrifuging the cell lysates (11,000 ×g, 15 min, 4°C). Then, Ac-DEVD-pNA buffer (15 μl) was added into the supernatants and the mixtures were incubated for 2.5 h. Finally, the absorbance (at OD405nm) was examined by a microplate reader apparatus.

### Recombinant lentivirus infection

Recombinant lentivirus for targeting KTN1-AS1 (sh-KTN1-AS1#1, sh-KTN1-AS1#2) and control lentivirus (sh-control) were generated by Weizhen Biological corporation (Jinan, Shandong, China). NSCLC cells were cultured in twenty-four-well plates with complete media and allowed to grow to 50-60% cell confluent. Then, the plates were changed with 300 μl (per well) fresh complete media and 25 μl (per well) lentivirus was added into the cells. The plates were kept at 37 °C with 5% CO_2_ for 12 h. Afterwards, the media were changed using fresh complete media and the cells were continue to be cultured until use.

### Xenograft assay

Twenty-four BALB/c nude mice (male; 5 weeks) were bought from Shanghai SLAC corporation (Songjiang, Shanghai, China). The mice were divided into three groups (eight mice/group): sh-control group, sh-KTN1-AS1#1 group and sh-KTN1-AS1#2 group. Then, a total of 1 × 10^7^ A549 cells (mixed with Matrigel; Total volume: 100 μl) which were stably infected with sh-control, sh-KTN1-AS1#1 or sh-KTN1-AS1#2 lentivirus, were subcutaneously injected into the left armpit of the mice. The diameters (long and wide) of the tumors in mice were recorded every four days. The mice were sacrificed four weeks after the initiation of cell injection, and the weight and volume of tumors in each group were evaluated. Tumor volumes were calculated according to the following formula: 0.5 × Long × Wide^2^. The xenograft assays were approved by the Committee on Animal Welfare of Binzhou Medical University Hospital.

### Wound-healing assays

Twelve hours post-transfection, NSCLC cells were trypsinized, washed by PBS and then re-plated into twelve-well plates with high density. The cells were allowed to grow to about 100% cell confluent. Subsequently, the wounds on cell monolayers were generated using pipette tips (200 μl). After washing the dead cells and cellular debris, the wound closures were photographed and recorded at 0 h and 48 h after the wounds were created.

### Transwell assay

NSCLC cell invasive capabilities were assessed by using Millipore transwell inserts (8 μm; Shanghe, Taizhou, Jiangsu, China). The inserts were pre-coated with Matrigel and put into twenty-four-well plates. After transfection (24 h later), NSCLC cells were digested and collected in a centrifuge tube. The cells were washed using PBS and counted. Subsequently, the cells (1.5 × 10^5^ cells per well) were placed into 250 μl media (without FBS), and the mixtures were subsequently placed into the inserts. Thereafter, 650 μl media (with 15% FBS) were added into the bottom of twenty-four-well plates. Then, the plates were kept at 37 °C with 5% CO_2_. After 24 h, the invaded cells were treated with methanol (1 ml) and crystal violet (0.1%). After treatment for 20 min, the cells were washed by PBS, and the photographs of the invaded cells were taken by a microscope.

### ChIP analysis

We used Millipore EZ ChIP analysis kits (Kaihong, Qingdao, Shandong, China) to carry out ChIP assays according to the kits’ protocols. Briefly, A549 cells maintained in 6 cm dishes were treated with formaldehyde (1%, 15 min, 27 °C). Then, glycine (125 nM) was added into the cells. After treatment, the cells were treated with cell lysis buffer. Subsequently, the 200 to 400 bp DNA fragments were obtained by sonicating the cell lysates. The anti-STAT1 antibody (Abcam, Cambridge, MA, USA) was then used for precipitating the DNAs. The normal rabbit IgG (Abcam, Cambridge, MA, USA**)** was used as a negative control. The precipitated DNA was eluted and subjected for real-time PCR analysis.

### RNA pull-down assays

We purchased the biotinylated RNAs (biotin-NC, biotin-KTN1-AS1) from Guangzhou Ruibo Biotechnology corporation (Guangzhou, Guangdong, China). Then, NSCLC cells were collected and treated with lysis buffer (supplemented with RNase inhibitor). Subsequently, the lysates were centrifuged with 12,000 ×g at 4 °C for 12 min. Then, we collected the supernatants. Afterwards, biotin-NC (200 pmol) or biotin-KTN1-AS1 (200 pmol) was added into the above supernatants for incubation (4 °C, 1.5 h). Then, Pierce streptavidin-agarose beads (100 μl; Ruibo, Guangzhou, Guangdong, China) into the above complexes, and they were incubated for 50 min at 4 °C. Finally, the miR-23b was purified and its foldchanges were examined by qPCR assays.

### Subcellular fractionation assay

The subcellular localization of KTN1-AS1 was determined by subcellular fractionation assays. The nuclear and cytoplasmic fractions were isolated by using the PARIS kits (Thermo, San Jose, CA, USA) in accordance with the kits’ instructions. In short, NSCLC cells were digested and collected in 500 μl fractionation buffer. The mixtures were incubated (4 °C, 20 min), followed by being centrifuged (500 ×g/min, 5 min, 4°C). Afterwards, the supernatants (cytoplasmic fractions) were collected for RNA extraction. The pellets (nuclear fractions) were treated using disruption buffer and used for RNA isolation. Then, the expression of KTN1-AS1 from the cytoplasmic and nuclear fraction was determined by qPCR. HPRT and U2 acted as the cytoplasmic and nucleus control, respectively.

### Luciferase reporter assay

KTN1-AS1 sequence involving wild-type or mutant-type miR-23b binding site was cloned into pGL3 empty luciferase reporter vector to generate luciferase reporters (KTN1-AS1 wild-type, KTN1-AS1 mutant-type). In addition, DEPDC1 mRNA 3’UTR sequence involving wild-type or mutant-type miR-23b binding sites were also respectively constructed into pGL3 empty luciferase reporter vector to generate luciferase reporters. The wild-type (wt) luciferase reporters were separately named as DEPDC1 wt1, DEPDC1 wt2 and DEPDC1 wt3; the mutant-type (mut) luciferase reporters were respectively named as DEPDC1 mut1, DEPDC1 mut2 and DEPDC1 mut3. Moreover, the wild-type or mutant-type sequence containing predicting binding 2 site (P2) of STAT1 in KTN1-AS1 promoter was also cloned into pGL3 vector, and the reporter was respectively named as P2 wt or P2 mut. NSCLC cells were co-transfected with: P2 wt or P2 mut reporters with ov-STAT1 or pcDNA3.1 empty plasmid; KTN1-AS1 wild-type or KTN1-AS1 mutant-type reporters with miR-23b mimics or NC mimics; Corresponding DEPDC1 wild-type or mutant-type reporters with miR-23b mimics or NC mimics. The pRL-TK plasmids were used as a normalizing control. The luciferase activities were determined by using Promega luciferase detection kits (Haifeng, Qingdao, Shandong, China) according to the kits’ protocols.

### Statistical analyses

SPSS software (version 19.0, Chicago, IL, USA) was applied for statistical analyses. Student’s t-test was employed to analyze the differences between two groups. One-way ANOVA was used to compare data containing more than two groups. Overall survival was assessed using the Kaplan-Meier methods and log-rank test. The dates of survival were evaluated by univariate and multivariate Cox proportional hazards models. A P-value less than 0.05 was considered statistically significant.

## Supplementary Material

Supplementary Figures
