# Supplementary material for: STAT1-induced upregulation of lncRNA KTN1-AS1 predicts poor prognosis and facilitates non-small cell lung cancer progression via miR-23b/DEPDC1 axis
Source: Aging (Albany NY). 2020 May 12;12(9):8680–701. doi: 10.18632/aging.103191 (PMC7244022; doi:10.18632/aging.103191)
Supplement: Supplementary Figures [file aging-12-103191-s001..pdf]

SUPPLEMENTARY FIGURES

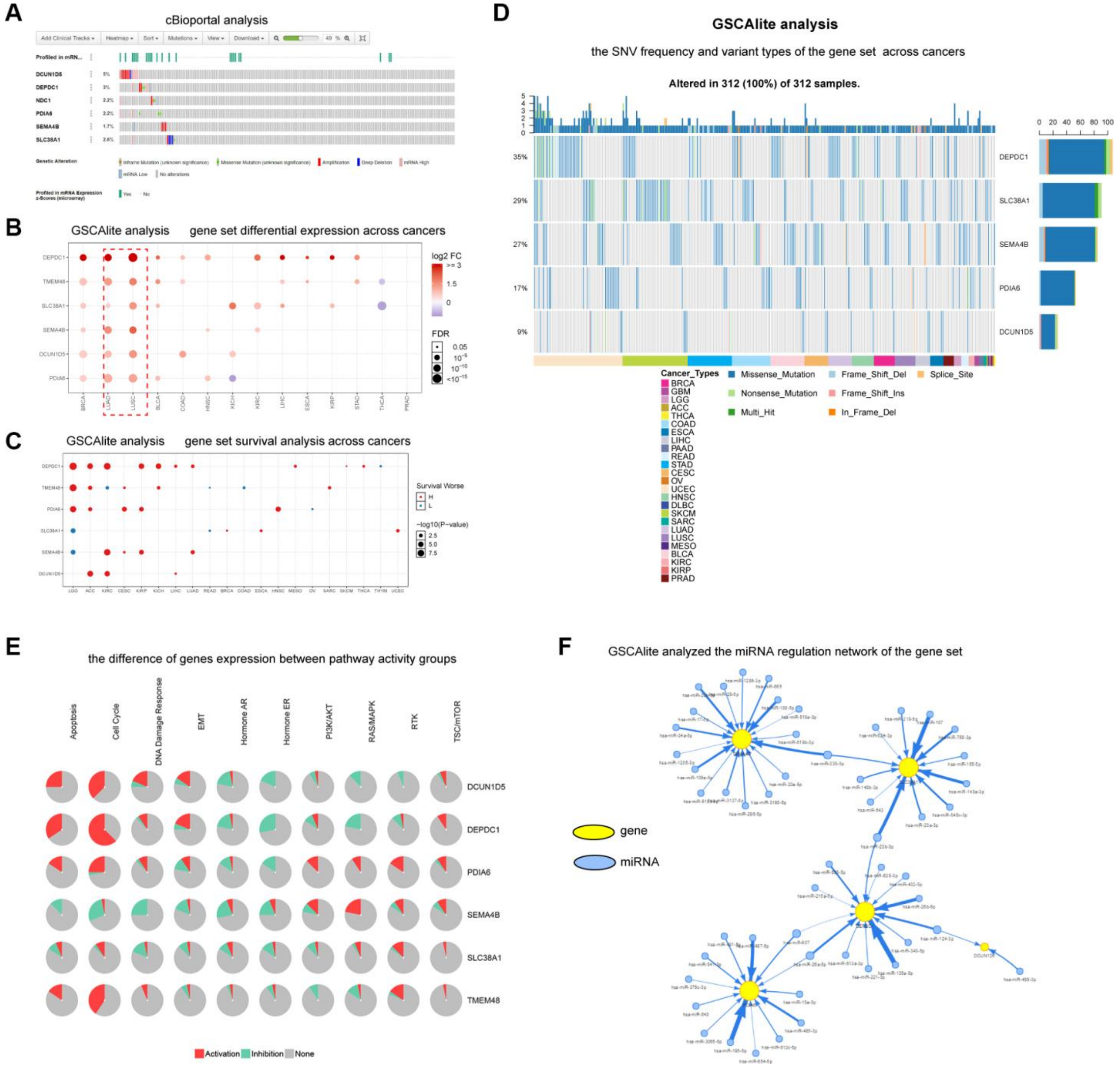

Supplementary Figure 1. (**A**) cBioportal program to analyze the genetic changes of these 6 genes. (**B**) GSCAlite algorithm analyzed the DEPDC1 expression across diverse cancer types. (**C**) GSCAlite algorithm analyzed the overall survivals relevant with DEPDC1 across diverse cancer types. (**D**) The SNV frequency of the 6 genes across cancers was analyzed by GSCAlite algorithm. (**E**) The pathway activity of the 6 genes was analyzed by GSCAlite algorithm. (**F**) GSCAlite algorithm was used to analyze the miRNA regulation network of these 6 genes.

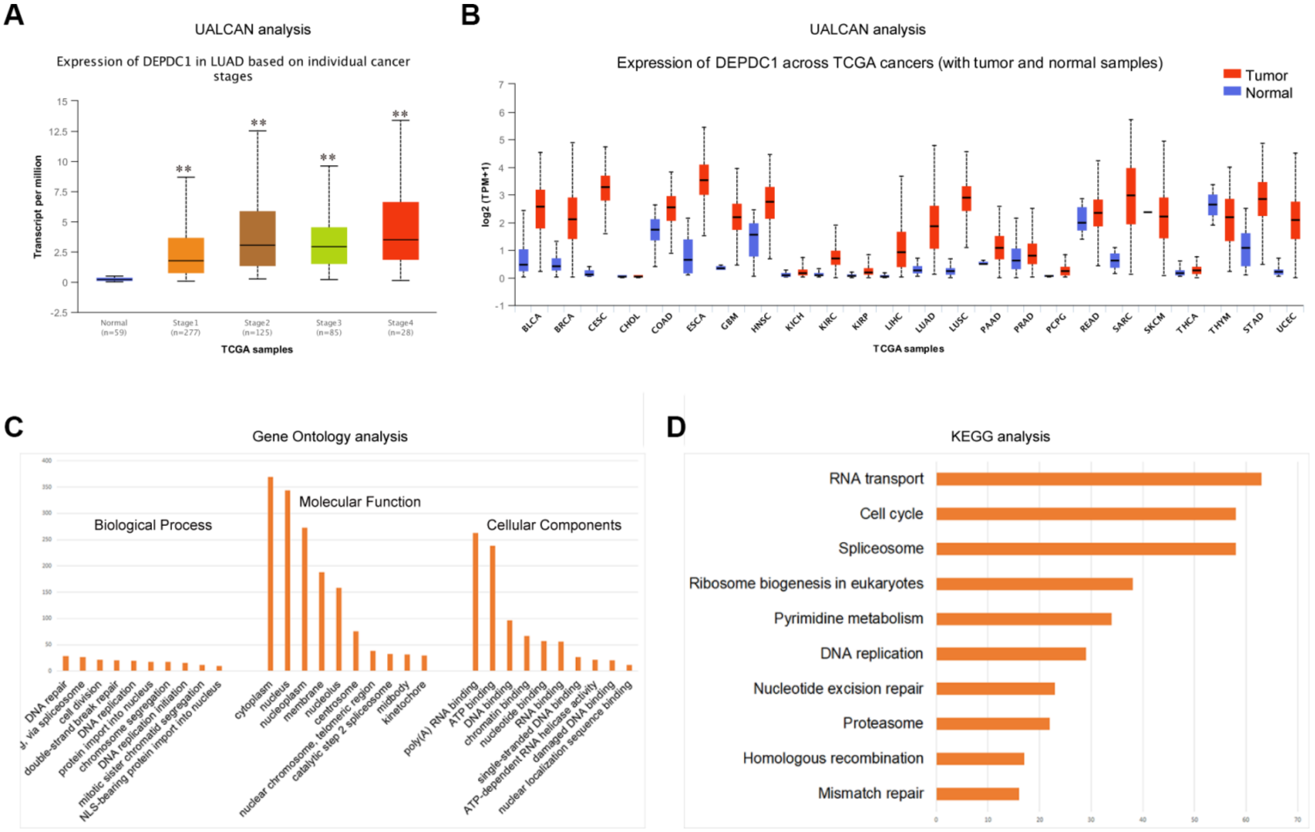

Supplementary Figure 2. (**A**) UALCAN program analyzed the DEPDC1 expression in stages of NSCLC using TCGA data. (**B**) UALCAN program analyzed the DEPDC1 expression across TCGA cancer types. (**C**) The genes positively correlated with DEPDC1 in lung cancer were obtained by using UALCAN program analysis, and GO analysis of these genes were conducted. (**D**) KEGG pathway analysis of the above genes. * $P < 0.05$, ** $P < 0.01$.
